# The role of intersection and street design on severity of bicycle-motor vehicle crashes

**DOI:** 10.1136/injuryprev-2016-042045

**Published:** 2016-11-09

**Authors:** Morteza Asgarzadeh, Santosh Verma, Rania A Mekary, Theodore K Courtney, David C Christiani

**Affiliations:** 1 Department of Environmental Health, Harvard T.H. Chan School of Public Health, Boston, Massachusetts, USA; 2 Center for Injury Epidemiology, Liberty Mutual Research Institute for Safety, Hopkinton, Massachusetts, USA; 3 MCPHS University, Boston, Massachusetts, USA

**Keywords:** Outcome of Injury

## Abstract

**Background:**

Safety concerns are a major barrier to cycling. Intersection and street design variables such as intersection angles and street width might contribute to the severity of crashes and the safety concerns. In this study we examined whether these design variables were associated with bicycle-motor vehicle crashes (BMVC) severity.

**Methods:**

Using the geographical information system and latitudes/longitudes recorded by the police using a global positioning device, we extracted intersection angles, street width, bicycle facilities, posted speed limits and annual average daily traffic from 3266 BMVC data from New York City police records. Additional variables about BMVC, including age and sex of the bicyclist, time of the day, road surface conditions, road character, vehicle type and injury severity, were obtained from police reports. Injury severity was classified as severe (incapacitating or killed) or non-severe (non-incapacitating, possible injury). The associations between injury severity and environment design variables were examined using multivariate log-binomial regression model.

**Findings:**

Compared with crashes at orthogonal intersections, crashes at non-orthogonal intersections had 1.37 times (95% CI 1.05 to 1.80) and non-intersection street segments had 1.31 times (95% CI 1.01 to 1.70) higher risk of a severe injury. Crashes that involved a truck or a bus were twice as likely to result in a severe injury outcome; street width was not significantly associated with injury severity.

**Conclusion:**

Crashes at non-orthogonal intersections and non-intersection segments are more likely to result in higher injury severity. The findings can be used to improve road design and develop effective safety interventions.

## Introduction

Safety concerns are a major barrier to riding a bicycle in the USA,^1^ preventing individuals from gaining the significant health benefits associated with cycling.^2–4^ These concerns are not unfounded. According to the National Highway Traffic Safety Administration Traffic Safety Facts, there were more than 49 000 counts of bicyclist injuries in 2012, including 726 fatal crashes.^5^ Bicyclists run a higher risk of being injured in a crash compared with other road users.^6^ Wegman and Aarts^7^ made a calculation for fatalities and severely injured individuals and stated that the incompatibility factor (ie, casualties in the weakest party divided by casualties in the strongest party) in the bicycle:car ratio was equal to 150:1. In other words, for each fatality or severe injury to motor vehicle occupants, there were 150 such injuries to bicyclists in bicycle-motor vehicle crashes (BMVCs).^7^

The number of bicyclists commuting to work has increased from 1.7 billion (2001) to 4 billion (2009) globally and the USA has seen an increase of 80% in 70 of its largest cities.^8^ ^9^ Efforts to encourage the use of bicycles for commuting or for sports and leisure activities in many developed countries^10–12^ may be aided by devoting greater resources to the prevention of bicycling-related crashes.

Most epidemiological studies of bicycling-related injuries have used data collected by State Police Departments, Emergency Medical Services or hospital data acquired either directly or through a data registry.^12–19^ Prior studies have reported inclement weather, darkness with no streetlights, morning peak rush hour (6:00–9:59), speeding-involved (vehicle speeds above 48.3 km/hour (30 mph)), truck involved, intoxicated driver, bicyclist aged ≥55, and intoxicated bicyclist to be among factors that significantly increase the risk of fatal injuries to bicyclists in BMVCs.^20^

The geographical information system (GIS) facilitates the location of a crash on a map as geographical aggregate data.^21^ Hence, GIS can be used to assess built environment factors that are not collected by crash report templates but that could be associated with crash severity. Some of the built environment variables that have been examined using GIS in prior BMVCs and injury studies include the number of legs and diameter of the central island in roundabouts,^22^ three measures of connectivity (ie, intersection density, average block length and connected node ratio),^23^ street network measures, street characteristics (eg, number of lanes, shoulder width, median presence, painted median width), socioeconomic data, traffic flow information^24^ and distances to the cycle tracks (bike lanes physically separated from motor vehicle traffic).^25^ Other epidemiological studies have explored the most frequent patterns of crashes and have focused on the existence of bicycle facilities such as cycle tracks as the main environment-related factor.^25^ ^26^

Many built environmental variables such as street width and intersection angle have been studied in the context of MVCs. For example, a study suggested skewed intersections may be dangerous for motor vehicles and their occupants.^27^ However, few studies have examined these features in relation to BMVCs. Our study aims to provide the first empirical findings on the association between intersection angle, street width and severity of bicyclist injury in BMVCs.

Furthermore, prior studies suggest street variables such as width are predictors of traffic speed^28^ ^29^ and also pedestrian behaviour, that is, higher pedestrian volumes are observed on narrower streets^30^ and may affect the severity of bicycle crash injuries. Second, the riding behaviour of bicyclists may change prior to the actual intersection (eg, slowing down or preparing for a turn) and crashes that occur ‘within intersection proximity’ can be influenced by the design of the intersection.

In this study, we aimed to examine intersection angle and street width and study their possible associations with severity of injury to the bicyclist in BMVC. We also examined the associations between environmental variables included in the police report and the severity of injury to the bicyclist in BMVC.

## Methods

Data for BMVCs that occurred in New York City (NYC) during 2011 were obtained from New York State Department of Transportation which originated from police reports. A total of 3350 crash cases were obtained. The crash records included crash coordinates collected by the police at the crash site with a global positioning system (GPS) device. We geocoded the crash coordinates using the Universal Transverse Mercator Zone 18 North in ArcMap 10.1 (ESRI, New York, New York, USA). Geospatial data about each crash location were then extracted by overlaying additional datasets and conducting spatial analysis.

### Environmental variables obtained by GIS analysis

Multiple layers of publicly available geospatial data were downloaded from the websites of New York State Department of Information Technology, NYC Open Data and the website of NYC. The street centreline layer embedded in the street grid files was used to measure intersection angle. Street dimension information was used to measure street width at each crash location. The geographical information used for the analysis was from 2011, the same year as the crash data.

#### Orthogonal intersection, non-orthogonal intersection and straight street

The crashes occurring within a 20 m radius of the centre of an intersection were considered as ‘at or close’ to that intersection (irrespective of the intersection categorisation on the police reports). Thus, we defined a 20 m radius from the centre of an intersection as ‘within intersection proximity’ in the spatial analysis, which is a little larger than 15 m adopted in other transportation studies.^31^ ^32^ This approach was chosen to account for minor geospatial errors in recording the location data.

The street centreline file depicts each street with one line that is in the centre of the street. Each centreline is composed of several segments that were drawn by a cartographer when developing the centreline map. Usually, a real intersection is where segment end points for each intersection leg collide and merge. In order to measure the street angle at the intersection closest to a crash location, the closest street centreline to the crash location was identified using ArcMap's analysis tools and stored as separate information. Then, the second-closest street centreline to the crash location was identified and stored. Both first and second segments had to be within 20 m of crash location for the angle measurement. If the second segment was more than 20 m away, we assumed that the crash was not at the intersection. The two segments in the buffer (ie, closest and second-closest segments) were isolated for each crash in ArcMap (please see online supplementary appendix 1, for details of intersection angle calculation).

10.1136/injuryprev-2016-042045.supp1 supplementary appendix 1

Since a street centreline is often made of multiple connected segments, if the first-closest and second-closest street segments to a crash location were on the same street centreline after the 20 m buffer had been applied and the calculated angle was 180° (±5°), then the crash was classified as outside intersection proximity.

In the analysis, 85<×<95° was treated as a right angle with ±5° for possible measurement error. A straight line was identified when we had a 175≤×≤185 measurement for an angle, that is, 180° between two street segments with ±5° of potential measurement error. A non-orthogonal angle was 0<×≤85 or 95≤×<175 (ie, more or less than 90°). On a 95≤×<175 measured intersection angle, at least one of the neighbouring street angles on the same intersection will have a 0<×≤85 angle which can contribute to limited driver and bicyclist visibility.

We compared our angle calculation algorithm results with Google Maps mapping service for a randomly sampled 50 crash locations for a validity check; the results from our algorithm were consistent with those reported by Google Maps.

#### Street width measurement

In order to calculate street widths, geocoded addresses were used to identify the closest street centreline. Then the street centreline's ID was used for a classic *attribute join* with street width data (*attribute join* is a tool in ArcMap that merges two datasets using a mutual identifying variable such as case numbers). This provided a spreadsheet with the street width data for each crash location.

Street width was categorised as ≤100 ft or >100 ft. According to American Association of State Highway and Transportation Officials (AASHTO) guidelines for street width, each car lane requires 11 ft, width of street parking space is 10 ft and minimum width for bicycle lane is 4 ft.^33^ ^34^ Considering 8 ft for space in the middle of the street and around the curbs and 10 ft for a sidewalk,^35^ the width for a normal street with two car lanes in each direction and bike lanes, car parking spaces and sidewalks on both sides of the street will be about 100 ft.

#### Annual average daily traffic

Annual average daily traffic (AADT) data for street sections were collected from the official website of the New York State DOT.^i^ The data were then geocoded using its roadway begin/end descriptions. The data contained a unique identifier that was coordinate-based and assigned to the roadway for which the AADT data were collected. This allowed the segment to be mapped in GIS when referenced along with begin and end points. The geocoded map was then overlaid on our crash maps after creating a similar projection and the AADT of the closest street segments to crash locations were collected using a classic *attribute join*.

#### Posted speed limits

The shapefiles^ii^ of the posted speed limits were collected from Vision Zero Data Feeds of NYC.^iii^ After merging and projecting the maps, posted speed limits for each crash location were collected using an *attribute join* command.

#### Bicycle facilities

A shapefile map of the bicycle facilities was obtained from the Municipality Office of NYC. Each type of bicycle facility had a defined set of attributes and descriptions in the shapefile. We first added the shapefile to our crash maps and then created similar projections for both maps to have them correctly overlaid. We then joined the crash locations with bicycle facilities to extract the attributes of bicycle facilities specific to each crash location. We imported the results in form of a spreadsheet into SAS and recoded the bicycle facilities. A crash location was coded as having a bicycle facility if there was a protected bike path, a greenway path or any type of bike route or bike lane. A crash location was coded as having no bicycle facility if there was a link, stairs, a proposed plan for any bicycle facility or no bicycle facility.

### Variables from NYC police reports

#### Road surface condition

Categories in our analysis included dry, wet and snow/muddy/slush. Muddy, snow/ice, slush and flooded water were separate categories in the police data but we combined them into one category because they represent a surface condition different from normal dry or wet and there were too few cases in each of these separate categories.

#### Road character

This category describes the roadway character at the location of the incident based on the police officer's observation/interpretation. Categories include straight and level, straight and grade, straight at hillcrest, curve and level, curve and grade and curve and hillcrest. Because of the small number of crashes in categories other than straight and level, road character was categorised into two categories: straight and level and the others.

#### Time of the day

A continuous variable in police data was categorised into morning (after 6:00–10:00), afternoon (after 10:00–15:00), evening (after 15:00–21:00) and night (after 21:00–6:00).

#### Vehicle type

The categories in the police reports included car, van, pickup truck, truck, bus and other vehicles. We combined these into three categories: car/van/pickup truck, truck and bus and other vehicles.

#### Age of the bicyclist

A continuous variable in the police data is the age of the bicyclist. We categorised it into children (6≤ age <18 years), young adults (18≤ age <40 years) and middle age and older adults (40≤ years).

#### Sex of the bicyclist

Categories included male and female which were not modified in our analysis.

### Outcome—severity of bicyclist injury

**Figure 1:**
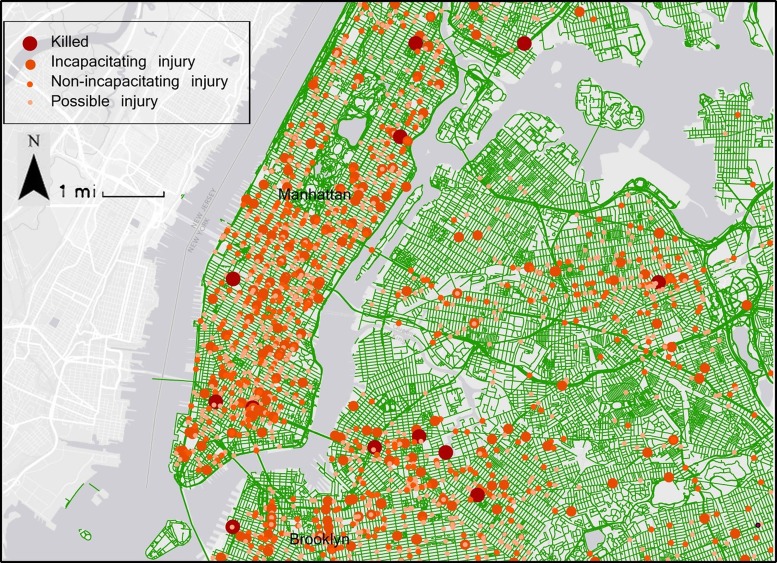
Distribution of bicycle-motor vehicle crashes in New York City in 2011.

Crash severity was obtained from the police reports (figure 1). The categories of ‘incapacitating injury’^iv^ and ‘killed’ were combined and coded as ‘severe injury’, while category of ‘non-incapacitating injury’^v^ and ‘possible injury’^vi^ were coded as ‘non-severe injury’.

If more than one person was injured in a crash and the data did not specify who among the injured was the bicyclist, we used the approach of Wei and Lovegrove^36^ and assumed the individual with the more severe injury was the bicyclist. This approach assumes that in a BMVC, the bicyclist is at a significantly higher risk of having a more severe injury than the vehicle occupants.

### Statistical analysis

Multivariate log-binomial generalised linear regression^37^ was used to examine the relationship between the risk of severe injuries and the built environment variables (eg, intersection angle and street width) and other variables obtained from the police reports. The log-binomial model provides risk ratio estimates for crash severity outcome. ORs, as calculated by logistic regression, are a good estimate of risk ratios when the outcome is rare. However, when the outcome is not rare, ORs overestimate risk ratios. Because the outcome in this study was not rare (11.2% of BMVCs resulted in a severe injury), we used the log-binomial model to estimate risk ratios for crash severity outcomes.^37^ The generic formula for the log-binomial regression model with a binary response y is log(π(x))=β_0_+β_1_x where mean chance of event is π(x).

Missing information was coded as ‘missing’ and included in the multivariate model. All the variables were included in the model regardless of their statistical significance. Risk ratios and the 95% CIs are presented. The analyses were conducted using SAS (V.9.4) software (SAS Institute, Cary, North Carolina, USA).

## Results

Information on injury severity was not available for 84 crashes and these cases were removed from the analysis. Of the remaining 3266 crashes, 367 resulted in severe bicyclist injuries (11.2%). We were able to calculate intersection angles for 2527 (77.4%) of the 3266 crash cases that occurred in NYC in 2011. Street width was calculated for all the crash sites. Table 1 shows the distribution of injury severity for intersection angle, street width, AADT, posted speed limits, bicycle facilities, road surface condition, road character, time of day, type of motor vehicle and age and gender of bicyclists. At least 1663 (1180+483,124, 72) BMVCs occurred at intersections (60%, for 22.6% intersection variable could not be coded). Majority of crashes occurred on streets with widths >100 ft (30 m), on a dry road surface condition, straight and level road and to male bicyclists.

**Table 1:** Population distribution of potential built environmental risk factors for bicycle-vehicle crash injuries according to the extent of injury, New York City, 2011

	Severity of bicycle-vehicle crash injury	
	Non-severe injury (non-incapacitating injury, possible injury) 2899	Severe injury (incapacitating injury or killed) 367
Intersection angle*		
Orthogonal (85<×<95)	1180 (90.5%)	124 (9.5%)
Non-orthogonal (0<×≤85 or 95≤×<175)	483 (87.0%)	72 (13.0%)
Straight street (175≤×≤180)	583 (87.3%)	85 (12.7%)
Missing	653 (88.4%)	86 (11.8%)
Width of street at crash location*		
≤30 m	890 (88.8%)	112 (11.2%)
>30 m	2009 (88.7%)	255 (11.3%)
Road surface condition		
Dry	2442 (88.3%)	324 (11.7%)
Wet/snow/muddy/slush	362 (90.5%)	38 (9.5%)
Missing	95 (95.0%)	5 (5.0%)
Road character		
Straight/level	2626 (88.7%)	335 (11.3%)
Straight/grade/hillcrest curve	175 (87.1%)	26 (12.9%)
Missing	98 (94.2%)	6 (5.8%)
Time of the day		
Morning (6:00–10:00)	390 (89.4%)	46 (10.6%)
Afternoon (10:00–15:00)	765 (89.0%)	95 (11.0%)
Evening (15:00–21:00)	1357 (89.8%)	154 (10.2%)
Night (21:00–5:00)	387 (84.3%)	72 (15.7%)
Vehicle type		
Car/van/pickup truck	2104 (89.9%)	236 (10.1%)
Truck and bus	90 (79.6%)	23 (20.4%)
Other vehicles	704 (86.7%)	108 (13.3%)
Age		
Children (6≤ age <18)	348 (89.0%)	43 (11.0%)
Young adults (18≤ age <40)	1518 (88.3%)	201 (11.7%)
Middle age and older (40≤ age)	635 (89.1%)	78 (10.9%)
Missing	398 (89.8%)	45 (10.2%)
Sex		
Male	2501 (88.9%)	312 (11.1%)
Female	377 (87.9%)	52 (12.1%)
Annual average daily traffic	2899 (88.8%)	367 (11.2%)
Posted speed limits	2899 (88.8%)	367 (11.2%)
Facilities		
Present	869 (87.9%)	120 (12.1%)
Not present	2030 (89.1%)	247 (10.8%)

*According to the ‘Methods’ section, it was not possible to measure the angle for all crashes due to the limitations of the geographical information system (GIS) data. Age, sex, vehicle type, time of day, road surface and road character are derived from the police reports, while intersection angle and width of streets at crash locations were measured using GIS techniques.

In the univariable log-binomial model, street width, road surface condition, road character, AADT, posted speed limits, bicycle facilities, age and gender of bicyclists were not significantly associated with the risk of having a severe injury for bicyclists who crashed with a motor vehicle (table 2). Non-orthogonal intersections and straight streets as compared with orthogonal intersections, truck and bus and other vehicles as compared with car/van/pickup truck and night time as compared with evening were associated with a higher risk of severe injury after a BMVC. In the multivariate model, the relationships between the above-mentioned variables and the risk of a severe injury did not change materially. The risk of a severe injury in crashes that occurred at non-orthogonal intersections (0<×≤85 or 95≤×<175) was 1.37 times (95% CI 1.05 to 1.80) higher than at orthogonal intersections (85<×<95 between the two streets). Also, crashes that happened outside the intersection proximity (ie, straight street segment—angle of 175≤×≤180) had 1.31 times (95% CI 1.01 to 1.70) higher risk of having a severe injury compared with crashes that occurred at orthogonal intersections (table 2). We also observed a significantly higher risk (RR=1. 54, 95% CI 1.19 to 1.99) of severe outcome in BMVCs that occurred during night as compared with those that occurred in the evening. When a truck or bus was involved in a BMVC, the injuries to bicyclist were twice as likely to be severe as compared with BMVC involvement of smaller cars, vans or pickup trucks.

**Table 2:** Risk ratios and CIs for the difference between severe (incapacitating injury or killed) and non-severe (non-incapacitating injury, possible injury) injuries for bicycle-vehicle crashes, New York City, 2011 (multivariable log-binomial generalised linear regression)

	Univariable RR (95% CI)	Multivariable RR (95% CI)*
Intersection angle		
Orthogonal (85<×<95)	1	1
Non-orthogonal (0<×≤85 or 95≤×<175)	**1.36 (1.04 to 1.79)**	**1.37 (1.05 to 1.80)**
Straight street (175≤×≤180)	**1.34 (1.03 to 1.73)**	**1.31 (1.01 to 1.70)**
Missing	1.22 (0.94 to 1.59)	1.19 (0.92 to 1.55)
Width of street at crash location†		
≤30 m	1	1
>30 m	1.01 (0.82 to 1.24)	0.94 (0.76 to 1.16)
Road surface condition		
Dry	1	1
Wet/snow/muddy/slush	0.80 (0.59 to 1.12)	0.77 (0.55 to 1.07)
Missing	0.43 (0.18 to 1.01)	0.38 (0.04 to 3.32)
Road character		
Straight/level	1	1
Straight/grade/ hillcrest curve	1.14 (0.79 to 1.66)	1.10 (0.76 to 1.58)
Missing	0.53 (0.23 to 1.12)	1.22 (0.19 to 7.94)
Time of the day		
Morning (6:00–10:00)	1.03 (0.76 to 1.41)	0.99 (0.73 to 1.35)
Afternoon (10:00–15:00)	1.08 (0.85 to 1.38)	1.03 (0.80 to 1.32)
Evening (15:00–21:00)	1	1
Night (21:00–5:00)	**1.54 (1.19 to 1.99)**	**1.54 (1.19 to 1.99)**
Vehicle type		
Car/van/pickup truck	1	1
Truck and bus	**2.02 (1.37 to 2.96)**	**2.11 (1.45 to 3.08)**
Other vehicles	**1.32 (1.06 to 1.63)**	**1.31 (1.06 to 1.63)**
Age		
Children (6≤ age <18)	0.94 (0.69 to 1.29)	0.99 (0.73 to 1.37)
Young adults (18≤ age <40)	1	1
Middle age and older (40≤ age)	0.94 (0.73 to 1.20)	0.93 (0.73 to 1.19)
Missing	0.87 (0.64 to 1.18)	0.94 (0.69 to 1.28)
Sex		
Male	1	1
Female	1.09 (0.83 to 1.44)	1.09 (0.83 to 1.44)
AADT	1.00 (0.99 to 1.01)	1.00 (0.99 to 1.01)
Posted speed limits	1.00 (0.99 to 1.01)	1.00 (0.99 to 1.01)
Facilities		
Present	1	1
Not present	0.89 (0.73 to 1.10)	0.91 (0.74 to 1.12)

Statistically significant values are formatted in bold.

*All variables were included in the multivariable model in addition to annual average daily traffic (AADT) and posted speed limits.

## Discussion

Our study is among the first to compare bicyclist injury severity from BMVCs occurring at different intersection angles and we observed increased risks of severe injury for non-orthogonal intersections and straight street segments. GIS analysis enabled us to examine the association between intersection design and the severity of BMVC and provided insights for possible ways to reduce crash severity. The built environment variables collected by the police at the crash scene (ie, road surface condition, road character) were not significantly associated with the severity of BMVC. Other variables including ‘truck and bus’ and ‘night time’ were associated with a higher risk of severe injuries after BMVC.

Police data are collected on templates that are designed primarily for vehicle-vehicle and vehicle-pedestrian crashes.^38^ The templates are not well designed to capture bicycle-specific variables that can inform built environment intersection design. For example, the information about bicyclists' location on a street is often recorded under ‘pedestrian's location’. Information about the built environment such as intersection configuration has been absent from police crash report templates. Our findings suggest that the built environment variables not reported on police templates may play a role in crash or injury severity. Including such variables on police templates has the potential to better inform street and intersection design.

We observed that a majority of crashes (60%) occurred at or close (within 20 m) to intersections (table 1). Other studies have also reported significantly higher rates of bicycle-vehicle collisions around traffic signals, bus stops and intersections^36^ (eg, up to 64% in Palo Alto, California, USA).^39^ Bicycle facilities such as cycle tracks in the USA do not typically continue through an intersection, although a significant proportion of BMVCs occur at intersections.^36^ ^39^ One could argue that effective bicycle facilities such as cycle lanes may impact only the crashes that occurred at non-intersection locations, where crashes could be less likely. Nevertheless, the results of our study provide evidence that although crashes at non-intersection locations may be less frequent than at intersections, straight streets are associated with a higher injury severity. A few studies have shown significant correlations between the speed of vehicles and the severity of injuries.^39^ The lower injury severity of intersection crashes could, therefore, be due to bicyclists’ and drivers' lowering their speed as they approach intersections. Cycle lanes at non-intersection locations could help reduce the risk of crashes resulting in both severe and non-severe injuries.^20^

We found non-orthogonal intersections to be associated with a higher risk of severe bicyclist injuries. A non-orthogonal intersection may allow limited visibility to bicyclists and drivers compared with orthogonal intersections. When the angle between two streets at an intersection is obtuse (providing better visibility), the other angle at the same intersection is typically acute (providing limited visibility). It is not clear whether the acute or the obtuse angle is associated with a higher injury severity. Drivers and bicyclists may have less time to prepare to react to possible hazards at an acute angle turn in a non-orthogonal intersection due to limited visibility. Other studies have reported an observed delay in reaction at non-orthogonal intersections which could partially explain this phenomenon.^40^ Shortening the time needed to prepare for entering an intersection has been associated with more severe injuries.^26^ ^41^ ^42^ Summala *et al*^43^ studied drivers' scanning behaviour at a T-intersection, particularly right turning drivers and concluded that speed reduction measures such as speed humps near intersections can improve drivers' visual search patterns in favour of the bicyclists coming from the right. Other studies have also found that speed reduction at intersections may improve hazard detection.^40^ ^44^ However, it is possible that travelling speed may be higher in obtuse intersections, causing higher impact crashes. Future research could examine driver and bicyclist behaviour patterns including slowing and braking and scanning for other vehicles at non-orthogonal intersections to understand the causal mechanisms by which intersection design can impacts severity of injuries in BMVCs.

Our research findings about the danger of non-orthogonal intersections in BMVCs as well as the limits of protection from bicycle facilities with respect to severity of injuries in BMVCs can have practical implications for practitioners and experts responsible for developing guidelines (eg, AASHTO and traffic engineers). Safety interventions and design modifications in non-orthogonal intersections (eg, installing bicycle traffic light) may reduce risk of severe injuries to bicyclists in BMVCs and thus needs further investigation. Also, it may be worthwhile to advocate for orthogonal intersections when planning for bicycle-friendly infrastructure. Additionally, we can prioritise non-orthogonal intersections when installing bicycle safety interventions.

Additional research is also needed to explore the reasons for the higher number of bicycle crashes at intersections compared with crashes outside the intersection proximity, with an eye to possible interventions. Although bicyclists are supposed to obey the same rules as motor vehicle drivers (ie, both must similarly stop at the red light), this may not always happen. Bicyclists often do not wait for a red light to change when there is no vehicular traffic.^45^ Some future topics to study are as follows: (1) Does the unpredictability of bicyclists' behaviour (such as not waiting for green lights) decrease the accuracy of drivers' predictions regarding bicyclists' behaviour and increase chances of crashes? (2) Does the length of the yellow light give bicyclists adequate time for stopping? (3) Can education in schools on the proper operation of bicycles on the road or licensing of bikes be a way to counteract unpredictability? (4) Would adjusting the vehicle-oriented (versus bicycle-oriented) traffic signal design at intersections in US cities to accommodate bicyclists increase safety? Some countries like the Netherlands have explored the use of traffic signal lights that turn green when groups of bicyclists approach or have lights for bicycles at intersections similar to pedestrian ‘walk’ lights. Increasing predictability of bicyclists' behaviour could greatly help bicyclists and drivers avoid collisions at intersections.

### Study limitations and strengths

This study uses a large number of BMVC data from the NYC to examine the associations between built environment variables and the risk of severe injury. The GPS data and the GIS analysis provided a new method to accurately measure the intersection angles for a large number of crashes. In our analysis we were able to examine only two built environment variables that could contribute to severity of injuries in crashes (ie, width of streets at crash locations and intersection angle). Other design elements should be considered in future research, such as lighting levels, to enhance the understanding of the role of the built environment and its effective modifications. In addition, we did not have information on important variables such as helmet use, traffic operations and controls, presence of street trees, actual speeds and on-street parking to include in our models.

Using GIS analysis, intersection angle could not be measured for 759 crash locations (ie, gave an error), which accounted for approximately 22% of the available cases. The majority of these errors occurred because the end points of street segments of an intersection did not match (see online supplementary appendix 1, Figure 1 and Figure 2 for measurement errors). This error was unlikely to be related to the severity of injury. To confirm, we used Google Maps to estimate angle measures of crash locations where ArcMap failed to provide an accurate measurement. We then repeated the log-binomial regression modelling with no missing values for street angle. The resulting effect estimates (ie, risk ratios) were farther away from the null and more significant. In the new multivariate modelling, the risk of a severe injury in crashes that occurred at non-orthogonal intersections (0<×≤85 or 95≤×<175) was 1. 55 times (95% CI 1.23 to 1.97) higher than at orthogonal intersections (85<×<95 between two streets). Also, crashes that happened outside the intersection proximity (ie, non-intersection street segment—angle of 175≤×≤180) had 1.39 times (95% CI 1.11 to 1.74) higher risk of having a severe injury compared with crashes that occurred at orthogonal intersections.

The bicycle count data are either non-existent or not very accurate in the USA and our inability to assess crash risk due to the lack of denominator data is another limitation of our study. Our results do not have interpretations for risk of BMVCs. For example, if a design feature significantly increases the risk of non-severe injuries and has no effect on the risk of severe injuries as compared with another design feature, then the proportion of severe injuries to all injuries (severe plus non-severe) associated with the former design feature will be lower, but the risk of all injuries would be higher. Last, our study used crash data from NYC, hence the results may not be generalisable to rural environments.

## Conclusion

Our study findings indicate that BMVCs at non-orthogonal intersections and non-intersection street segments (straight street) are more likely to result in a severe injury to bicyclist than BMVCs at orthogonal intersections. These findings suggest that non-orthogonal intersections and non-intersection street segments may warrant priority for protective interventions. Geospatial analysis provides a new avenue to study built environment factors in transportation research and may help in developing effective interventions to protect bicyclists on the road. Potential interventions for non-intersection street segments include protected bike lanes (ie, cycle tracks) which have been shown to reduce the risk of crashes. Since cycle tracks do not continue to physically divide the space and protect bicyclists at intersections in the USA, other possible interventions should be considered for regulating vehicular movement. Potential interventions for non-orthogonal intersections could include the installation of bicycle signals as part of the intersection signalisation. An intervention such as installation of bicycle signals may be more impactful on non-orthogonal intersections as compared with orthogonal intersections.

**What is already known on the subject**

- Bicycling yields great public health benefits and safety concerns are a major barrier to bicycling.
- Although a majority of bicycle-MVCs happen at intersections in the USA, protective bicycling infrastructure are not designed for intersections.
- Associations of intersection angle and street width with the severity of injury in bicycle-MVCs has not been examined.

**What this study adds**

- As compared with orthogonal intersections, non-orthogonal intersections and straight streets were found to be associated with a higher risk of severe injury after a bicycle-MVC.
- Crashes that involved a truck or a bus as compared with smaller vehicles were twice as likely to result in a severe injury.
- Geographical information system analysis provides a unique opportunity to examine the association between built environment factors and the risk of MVC injuries.

## Footnotes

i https://data.ny.gov/Transportation/Annual-Average-Daily-Traffic-AADT-Beginning-1977/6amx-2pbv.

ii Esri defines shapefile (.shp) spatial data format as one that stores non-topological geometry and attribute information for the spatial features in a dataset. The geometry for a feature is stored as a shape comprising a set of vector coordinates.

iii http://www.nyc.gov/html/dot/html/about/vz_datafeeds.shtml#speed.

iv According to the 2006 Fatality Analysis Reporting System (FARS) coding and validation manual, “an incapacitating injury includes any injury, other than a fatal injury, which prevents the injured person from walking, driving or normally continuing the activities the person was capable of performing before the injury occurred. This includes: severe lacerations, broken or distorted limbs, skull or chest injuries, abdominal injuries, and unconsciousness at or when taken from the crash scene, and unable to leave the crash scene without assistance. Does not include momentary unconsciousness”.

v “Any injury, other than a fatal injury or an incapacitating injury, which is evident to observers at the scene of the crash in which the injury occurred. This includes: lump on head, abrasions, bruises and minor lacerations. This does not include limping unless any actual injury can be seen”.

vi “A possible injury is any injury reported or claimed which is not a fatal injury, incapacitating injury or non-incapacitating evident injury. This includes: momentary unconsciousness, claim of injuries not evident, limping, complaint of pain, nausea and hysteria”.
