# Supplementary material for: The role of intersection and street design on severity of bicycle-motor vehicle crashes
Source: Inj Prev. 2016 Nov 9;23(3):179–85. doi: 10.1136/injuryprev-2016-042045 (PMC5502254; doi:10.1136/injuryprev-2016-042045)
Supplement: supplementary appendix 1 [file injuryprev-2016-042045supp001.pdf]

Appendix 1.

A Python script was developed in Arc MAP ™ 9.3.1 to measure the intersection angles. Step 1) A 20m buffer was drawn around each crash location and information outside the buffer zone was eliminated. Step 2) The Python script identified the closest (A) and the second closest street segments to the crash location (B) within the buffer. The Python script also identified the two ends of the segments A and B within the buffer, and then compared their X and Y against each other to find a match. If the X and Y of two spots were the same, that was defined as the intersection's center point. Step 3) The Python script measured the length of segments A and B using the formula presented in Figure 1. It also measured the distance between the other two end points of the segments A and B, and saved it as segment C. Step 4) The Python script used the following mathematic formula to calculate the angle of the intersection and saved the final measure in a separate file for our statistical analysis (Figure 1). The measurement was repeated for all 3350 crashes, and their output was recorded separately.

= degrees (acos((A * A + B * B - C * C)/(2.0 * B * C)))

Where A, B and C are the length of the sides of the triangle.

There were a few scenarios in which the angle measurement method did not provide an angle. For example, if a crash did not occur at an intersection but there was a second street line parallel to the first within the 20 meter buffer zone at the crash location. Since the two centerlines did not have a mutual point, ArcMap was not able to identify an intersection center and, thus, did not measure an angle (Figure 2, A). Having many streets close to each other could be a factor in increasing the risk of measurement error in this case. If the cartographer who

developed the centerlines did not completely join the ends of the two segments to each other at an intersection, causing different X and Y readings for each of the four end points (figure 2, B). The same scenario occurred if the cartographer put the end of a segment after the intersection center point and not exactly at the center point, this caused miscalculation because Python script was not able to find a mutual endpoint between the two segments (Figure 2, Ct). Finally, if at least one of the two identified street segments closest to the crash location had a significant change of direction within the 20 meter buffer zone, Python script could not calculate the angle because the measurement of one of the triangle sides was not realistic, thus giving a mathematical error in the formula's calculation of sides and angles (Figure 2, D). The above three error types occurred in 759 crash locations at random, resulting in a null value. These values were treated as "missing" in the statistical analysis.

In a four-way intersection with two 90 degree turns and one turn at greater than 90 degrees, there is a possibility that our spatial analysis method selects the two 90 degrees intersection segments. Such issue can underestimate the number of intersections with a non-orthogonal turn. We believe the chances of such errors are very low. However, such potential misidentification of closest two street segments should be considered by other scientists if they plan to apply our methodology to their research.
